# Applying the Multi-Theory Model of Health Behavior Change to Examine Depression Among U.S. Adults with Diagnosed Diabetes

**DOI:** 10.3390/healthcare14070875

**Published:** 2026-03-28

**Authors:** Farhana Khandoker, Manoj Sharma

**Affiliations:** 1 Department of Social and Behavioral Health, University of Nevada, Las Vegas, NV 89154, USA; manoj.sharma@unlv.edu; 2 Department of Internal Medicine, University of Nevada, Las Vegas, NV 89154, USA

**Keywords:** diagnosed diabetes, depression, multi-theory model, emotional distress, social determinants of health

## Abstract

**Background/Objectives:** Depression is a common and consequential comorbidity among adults with diagnosed diabetes. Prior research has largely emphasized individual health behaviors, with less attention to emotional burden, social context, or theory-driven interpretation. The Multi-Theory Model (MTM) of Health Behavior Change offers an integrative framework for examining behavioral, emotional, and environmental correlates of health outcomes. This study applied MTM to examine correlates of lifetime diagnosed depression among U.S. adults with diagnosed diabetes. **Methods:** This cross-sectional study analyzed 2023 Behavioral Risk Factor Surveillance System (BRFSS) data from 19,967 adults with diagnosed diabetes, representing approximately 30 million U.S. adults after survey weighting. Lifetime diagnosed depression was assessed based on respondents reporting that a health professional had told them they had a depressive disorder, representing a lifetime history of depression rather than current depressive symptoms. Independent variables were organized into behavioral, emotional, and environmental domains consistent with MTM. Survey-weighted descriptive analyses, Rao–Scott χ^2^ tests, and nested survey-weighted logistic regression models were conducted. **Results:** The weighted prevalence of lifetime diagnosed depression among adults with diagnosed diabetes was 24.3%. In the fully adjusted MTM-guided model, emotional and environmental domains showed the strongest associations with lifetime diagnosed depression. Frequent mental distress was associated with substantially higher odds of depression (adjusted odds ratio ≈ 10.4, *p* < 0.001). High social or economic stress and fair or poor self-rated health remained independently associated (*p* < 0.001). Behavioral factors, including physical activity, smoking, and body mass index, were attenuated after adjustment. **Conclusions:** Lifetime diagnosed depression among adults with diagnosed diabetes was more strongly associated with emotional burden and adverse social conditions than with health behavior alone, supporting the integration of distress screening and context-responsive interventions into diabetes care.

## 1. Introduction

### 1.1. Burden of Depression in Diagnosed Diabetes

Depression is a frequent and clinically meaningful comorbidity among adults living with diagnosed diabetes. Population-based evidence consistently demonstrates that adults with diabetes experience substantially higher rates of depression than adults without diabetes. For example, an analysis of BRFSS data from 2011 to 2019 found that the prevalence of diagnosed depression among U.S. adults with diabetes was markedly higher than among adults without diabetes, reaching 29.2% in 2019 among adults with diagnosed diabetes [1]. These findings are consistent with systematic reviews and large epidemiologic studies showing substantially elevated depression prevalence among adults with diabetes across diverse populations [2,3] Beyond prevalence, longitudinal evidence suggests a bidirectional relationship between depression and diabetes, with each condition increasing the risk of the other over time [3]. This comorbidity is clinically significant because depression can interfere with the daily demands of diabetes self-management. Individuals experiencing depressive symptoms often report greater difficulty engaging in behaviors that support glycemic control, including physical activity, medication adherence, healthy eating, and smoking cessation [4]. Depression may also increase perceived disease burden, reduce motivation, and impair emotional and cognitive resources needed for sustained self-care. From a public health perspective, the co-occurrence of depression and diabetes is associated with increased functional disability, higher health care utilization, and poorer health outcomes. Given the high and rising prevalence of diabetes in the United States, the population-level impact of comorbid depression is substantial. Systematic reviews further confirm that depression remains highly prevalent among individuals with diabetes globally, underscoring the importance of identifying modifiable psychosocial and contextual drivers of risk [5].

### 1.2. Gaps in Existing Research

Despite extensive evidence documenting the high prevalence of depression among adults with diabetes, important gaps remain in how this relationship has been conceptualized and examined. Much of the existing literature has focused primarily on individual health behaviors such as physical activity, body weight, and smoking when investigating depression in diabetes populations [6,7]. Although these behavioral factors are relevant, examining them in isolation may overlook the broader emotional and environmental conditions that shape depression risk. Growing evidence indicates that psychosocial stress, diabetes-related distress, and social determinants of health are important influences on mental health outcomes among adults with diabetes [8,9]. Another limitation of the literature is the reliance on clinic-based samples or disease-specific cohorts, which may limit generalizability and underrepresent individuals experiencing socioeconomic disadvantage or barriers to care [10,11]. While such studies provide important clinical insights, they may not capture population-level patterns of depression across diverse demographic and socioeconomic groups. Population-based analyses using nationally representative datasets are comparatively less common and often prioritize descriptive associations rather than theory-informed interpretation. Relatively few studies explicitly apply contemporary behavior change theory to organize the examination of depression as a primary outcome in adults with diabetes. In many analyses, depression is treated as a covariate, mediator, or secondary outcome rather than a central phenomenon requiring theoretical explanation [12,13]. Without a guiding conceptual framework, behavioral, emotional, and environmental influences are frequently examined independently rather than as interconnected domains within a broader system. This fragmentation limits the ability to determine which domains may be most actionable for public health practice. Applying a structured and theory-driven framework may therefore provide clearer insight into how behavioral practices, emotional processes, and environmental contexts interact to shape depression risk among adults with diagnosed diabetes.

### 1.3. Overview of the Multi-Theory Model (MTM)

The MTM of Health Behavior Change is a contemporary, fourth-generation, integrative framework designed to synthesize key constructs from multiple behavior change theories into a single pragmatic model that distinguishes between processes involved in initiating change and those required to sustain change over time [14]. This distinction is particularly relevant for complex and chronic health outcomes, such as depression among adults with diagnosed diabetes, where short-term behavior modification may not translate into long-term well-being without attention to emotional regulation and environmental context. MTM’s emphasis on both initiation and sustenance processes allows for a more comprehensive understanding of health outcomes shaped by ongoing stressors, emotional burden, and social constraints rather than by individual behavior alone [15]. Within MTM, the initiation of change is influenced by participatory dialogue, behavioral confidence, and changes in the physical environment. Participatory dialogue refers to a balanced cognitive appraisal of the advantages and disadvantages of adopting health-related behavior, with change more likely when perceived advantages outweigh perceived disadvantages. Behavioral confidence reflects an individual’s perceived ability to initiate a behavior despite challenges and barriers and is conceptually related to, but distinct from, traditional notions of self-efficacy. Changes in the physical environment refer to the availability and accessibility of tangible resources that facilitate behavior initiation, such as safe spaces for physical activity, access to healthy foods, or access to health services.

Although participatory dialogue and behavioral confidence are typically measured directly in intervention-based studies, population surveillance data such as the BRFSS captures related behavioral expressions that reflect initiation processes. In the present study, behavioral factors measured using BRFSS variables included physical activity participation, smoking status, alcohol use, and body mass index. These variables reflect engagement in or avoidance of health-related behaviors that are often targeted in diabetes self-management programs and are commonly examined as correlates of depression in prior research [6,16]. Within the MTM framework, these behaviors represent observable manifestations of initiation-related processes but do not fully capture the sustaining forces that shape long-term mental health outcomes. Importantly, this study does not test MTM constructs directly nor evaluate the full causal structure of the model. Instead, MTM is applied as a conceptual heuristic to organize epidemiologic inquiry and guide the selection and interpretation of behavioral, emotional, and environmental correlates of lifetime diagnosed depression within population-based surveillance data.

Sustenance of change within MTM is supported by emotional transformation, practice for change, and changes in the social environment. Emotional transformation refers to the ability to direct and regulate emotional experiences in ways that support continued engagement in health-related functioning despite adversity. In this study, emotional transformation was operationalized using the BRFSS measure of frequent mental distress, defined as experiencing 14 or more mentally unhealthy days in the past 30 days. Although frequent mental distress serves as a useful indicator of sustained emotional strain within the MTM framework, it also reflects experiences that partially overlap with depressive symptomatology. Mentally unhealthy days capture recent emotional difficulty and psychological burden that may occur alongside clinically diagnosed depression. Consequently, within this analysis, the measure is interpreted primarily as a population-level signal of ongoing emotional distress rather than a completely independent causal determinant of depression. This indicator captures persistent emotional strain and diminished emotional regulation, which are central to understanding depression risk among adults managing the ongoing demands of diagnosed diabetes [17]. Frequent mental distress provides a population-level proxy for compromised emotional transformation, aligning closely with MTM’s emphasis on emotional processes as sustaining mechanisms.

Practice for change, also referred to as ongoing practice, denotes the deliberate and sustained repetition of health-related behaviors over time, reinforcing habit formation and supporting long-term maintenance beyond initial adoption. Changes in the social environment refer to contextual conditions that either facilitate or undermine an individual’s capacity to maintain health-related functioning, including social support, socioeconomic stability, and access to community resources.

**Figure 1:**
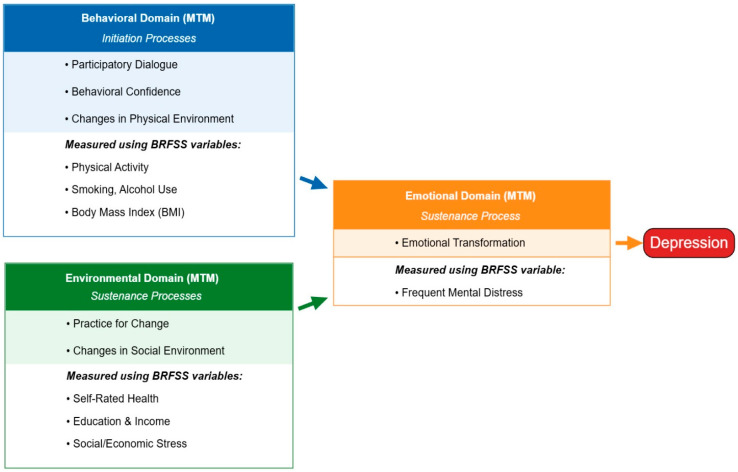
MTM-guided conceptual framework illustrating behavioral, emotional, and environmental domains associated with depression among adults with diagnosed diabetes.

In the present analysis, environmental context was operationalized using BRFSS measures of self-rated general health, educational attainment, household income, health insurance coverage, and social or economic stress related to difficulty meeting basic needs. These variables reflect structural and contextual conditions that shape access to resources, exposure to chronic stress, and perceptions of health and control. Prior research has consistently shown that social and economic stressors are strongly associated with depression among adults with diabetes, often exerting effects that persist even after accounting for individual behaviors [18,19]. Depression among adults with diagnosed diabetes reflects a complex interplay of behavioral demands, sustained emotional distress, and environmental strain. Managing diabetes requires ongoing self-regulation in the context of fluctuating symptoms, treatment burden, and social or economic pressures, making MTM particularly well suited for organizing these interrelated influences. By applying MTM to nationally available BRFSS data, this study moves beyond isolated examination of behaviors to provide an integrated, theory-informed assessment of how behavioral, emotional, and environmental domains jointly shape depression risk. In this framework, behavioral practices and environmental conditions are conceptualized as relating to depression partly through sustained emotional burden and emotional regulation capacity. As illustrated in Figure 1, the emotional domain is shared by both behavioral and environmental domains because emotional transformation represents the primary sustaining pathway through which behaviors and contextual conditions shape depression risk among adults with diagnosed diabetes.

### 1.4. Study Objective

Given the need for theory-informed, population-level evidence that integrates behavioral, emotional, and environmental influences on mental health in chronic disease, this study seeks to advance the understanding of lifetime diagnosed depression among adults with diagnosed diabetes from a public health perspective. Using the MTM of Health Behavior Change as an organizing framework, this analysis examines how behavioral practices, emotional distress, and environmental conditions are associated with a lifetime history of diagnosed depression among adults with diagnosed diabetes. By applying MTM to recent BRFSS data, this study aims to provide an integrated empirical foundation that can inform context-sensitive approaches to mental health screening and support within diabetes care.

## 2. Methodology

### 2.1. Study Design and Data Source

This study used a cross-sectional design based on 2023 Behavioral Risk Factor Surveillance System (BRFSS) data. The BRFSS is a nationally representative, state-based health survey conducted annually by the Centers for Disease Control and Prevention (CDC) in collaboration with U.S. states and territories [20]. The survey collects information on health behaviors, chronic conditions, and access to health care among noninstitutionalized adults aged 18 years and older residing in the United States. Data are collected through landline and cellular telephone interviews using a complex multistage probability sampling design, allowing estimates to be generalized to the civilian, noninstitutionalized adult population [20]. Sampling weights were applied to account for unequal probabilities of selection, nonresponse, and post-stratification to U.S. population demographics, allowing estimates to be generalized to the civilian, noninstitutionalized adult population. Sampling weights were applied using the BRFSS final combined landline and cellular weight (_LLCPWT), along with the stratification variable (_STSTR) and primary sampling unit (_PSU), to account for the complex survey design. The 2023 BRFSS public-use dataset includes respondents from 48 states, the District of Columbia, Guam, Puerto Rico, and the U.S. Virgin Islands, as two states did not meet minimum public dataset requirements. Because this analysis was based on core questionnaire variables, appropriate core survey design variables and weights were applied. The 2023 BRFSS questionnaire, calculated variables documentation, and annual data overview were consulted to ensure accurate variable construction and reproducibility. The study relied exclusively on publicly available, de-identified BRFSS data and did not involve direct contact with human participants. Because the dataset contains no personally identifiable information and is accessible for public use, this study was exempt from institutional review board (IRB) review in accordance with federal regulations governing human subject research.

### 2.2. Study Population

The analytic sample included adults who reported being told by a health professional that they had diabetes based on the BRFSS item DIABETE4 (excluding respondents who reported gestational diabetes only or prediabetes). Because the BRFSS core questionnaire does not consistently distinguish the diabetes type across all states, the analytic sample should be interpreted as adults with diagnosed diabetes rather than definitively type 2 diabetes in all cases. Respondents who reported gestational diabetes only, prediabetes, or missing or indeterminate diabetes status were excluded. Additional exclusions were applied for missing responses on the primary outcome variable and key covariates included in the multivariable models. After applying all inclusion and exclusion criteria, the final analytic sample consisted of 19,967 unweighted respondents with diagnosed diabetes. When survey weights were applied, this sample represented approximately 30 million U.S. adults with diagnosed diabetes, reflecting the nationally representative civilian, noninstitutionalized adult population.

### 2.3. Measures

#### 2.3.1. Outcome Variable

The primary outcome was lifetime diagnosed depression, assessed using the BRFSS item ADDEPEV3. Respondents were asked whether a health professional had ever told them that they had a depressive disorder, including depression, major depression, dysthymia, or minor depression. This item is part of the BRFSS chronic health conditions section and reflects a lifetime diagnosis (“ever told”) of depressive disorder. Responses were coded dichotomously (yes/no). This measure reflects a lifetime history of diagnosed depression rather than current symptom severity. Because the timing of diagnosis relative to diabetes onset cannot be determined in BRFSS data, the variable is interpreted as an indicator of comorbid lifetime depression history among adults with diagnosed diabetes rather than a measure of current depression [20,21].

#### 2.3.2. MTM-Guided Independent Variables

Independent variables were selected and organized using the MTM of Health Behavior Change as a conceptual framework to distinguish between initiation and sustenance processes relevant to depression among adults with diagnosed diabetes [14,15]. Because BRFSS is a population surveillance system rather than an intervention-based dataset, MTM constructs were operationalized using theoretically aligned proxy measures rather than direct construct scales. In this study, MTM was applied as an organizing and interpretive framework to guide variable selection and model specification rather than as a direct test of individual constructs. Behavioral variables represented initiation-related processes within MTM. These included participation in leisure-time physical activity, smoking status, alcohol use, and body mass index. These indicators reflect engagement in or avoidance of health-related behaviors commonly targeted in diabetes self-management and are frequently examined as correlates of depression in prior epidemiologic research [6,16]. Within the MTM framework, these behaviors represent observable expressions of initiation processes occurring within individuals’ physical and social contexts.

Emotional sustenance within MTM is centered on emotional transformation, defined as the capacity to manage and redirect emotional experiences in ways that support continued functioning despite adversity. In this analysis, emotional transformation was approximated using the BRFSS measure of frequent mental distress defined as experiencing 14 or more mentally unhealthy days in the past 30 days, consistent with CDC BRFSS guidance and the calculated variable for frequent mental distress (_MENT14D) derived from the MENTHLTH item. This indicator captures persistent emotional burden and impaired emotional regulation and aligns closely with MTM’s conceptualization of emotional processes as central sustaining mechanisms influencing depression risk among adults managing the ongoing demands of diagnosed diabetes [7,22].

Environmental sustenance processes within MTM reflect the broader social and structural context that can either support or undermine sustained health outcomes. Environmental variables include self-rated general health, educational attainment, household income, health insurance coverage, and social or economic stress related to difficulty meeting basic needs. These indicators capture access to resources, exposure to chronic stress, and perceived control over health and daily life, all of which are central to MTM’s emphasis on the social environment as a determinant of sustained well-being [18,19]. A detailed mapping of BRFSS variables to MTM domains is provided in Table 1, and the MTM-guided conceptual framework is illustrated in Figure 1.

### 2.4. Statistical Analysis

All analyses accounted for the complex survey design of BRFSS by incorporating sampling weights, stratification variables, and primary sampling units (PSUs). Weighted descriptive statistics were calculated to summarize sample characteristics and to estimate the prevalence of lifetime diagnosed depression among adults with diagnosed diabetes. Bivariate associations between depression and categorical independent variables were examined using Rao–Scott χ^2^ tests, which adjust for the complex survey design [23]. For continuous variables, survey-weighted regression models were used. Multivariable associations were examined using nested survey-weighted logistic regression models. Collinearity diagnostics were examined before model estimation and did not indicate problematic multicollinearity among included predictors. Model 1 included sociodemographic covariates (age group, sex, and race or ethnicity). Model 2 added behavioral factors consistent with MTM initiation processes. Model 3 included behavioral, emotional, and environmental variables, representing the full MTM-guided model. Results are reported as adjusted odds ratios (AORs) with 95% confidence intervals (95% CIs). A sensitivity analysis was conducted in which BMI was modeled as a categorical variable rather than a continuous measure to assess the robustness of findings to an alternative specification of body weight. Statistical significance was evaluated at α = 0.05 (two-sided). All analyses were conducted using R (version 4.3.x) with appropriate survey analysis packages.

### 2.5. Reporting Guidelines

This study was conducted and reported in accordance with the Strengthening the Reporting of Observational Studies in Epidemiology (STROBE) guidelines for cross-sectional studies [24].

## 3. Results

### 3.1. Sample Characteristics

The analytic sample consisted of 19,967 adults with diagnosed diabetes, representing approximately 30 million U.S. adults after application of BRFSS survey weights. The weighted prevalence of lifetime diagnosed depression was 24.3%, indicating that nearly one in four adults with diagnosed diabetes reported having been diagnosed with depression at some point in their lives. The weighted sample was predominantly middle-aged and older, consistent with the epidemiology of diagnosed diabetes in the United States. Women and men were represented in nearly equal proportions. Most respondents reported having health insurance coverage, although a substantial proportion rated their general health as fair or poor. Behavioral risk factors were common, including physical inactivity, smoking, and alcohol use. A notable proportion of respondents reported frequent mental distress, underscoring the substantial emotional burden experienced by adults living with diagnosed diabetes [20].

### 3.2. Bivariate Associations

Survey-weighted bivariate associations between depression status and study variables are summarized in Table 2. Depression was significantly associated with multiple behavioral, emotional, and environmental factors at the bivariate level. A higher prevalence of depression was observed among respondents who reported physical inactivity, smoking, and alcohol use. Depression was also more prevalent among individuals reporting frequent mental distress, fair or poor self-rated general health, lower educational attainment, lower household income, lack of health insurance, and higher levels of social or economic stress. Sociodemographic characteristics, including age group, sex, and race or ethnicity, were also significantly associated with depression status. These findings indicate that depression among adults with diagnosed diabetes is patterned across behavioral practices, emotional well-being, and broader social and environmental contexts.

### 3.3. Multivariable MTM-Guided Models

Results from the survey-weighted logistic regression analyses are presented in Table 3. Model 3, which incorporates behavioral, emotional, and environmental domains, represents the full MTM framework, and is treated as the primary multivariable model for interpretation. Models 1 and 2 are presented as nested models to illustrate the sequential contribution of sociodemographic and behavioral factors prior to the inclusion of emotional and environmental variables. In the primary MTM model, emotional and environmental factors emerged as the most robust correlates of depression among adults with diagnosed diabetes. Frequent mental distress was strongly associated with higher odds of depression, even after adjustment for sociodemographic characteristics and health behaviors (adjusted odds ratio [AOR] > 10.0, *p* < 0.001). Indicators of adverse environmental context, including high social or economic stress and fair or poor self-rated health, also remained independently associated with depression in the fully adjusted model (*p* < 0.001). In contrast, several behavioral factors that were statistically significant in earlier models demonstrated attenuation after the inclusion of emotional and environmental domains. Physical activity, smoking status, and body mass index were no longer independently associated with depression in Model 3, whereas alcohol use remained modestly associated after full adjustment. This suggests that the association between health behaviors and depression may be partly accounted for by underlying emotional burden and contextual stressors. This pattern is consistent with the MTM framework, which emphasizes emotional transformation and environmental support as key sustaining mechanisms associated with long-term mental health outcomes [14,15]. Because frequent mental distress reflects recent emotional symptoms that may coincide with experiences of depression, this relationship should be interpreted as representing concurrent emotional burden rather than evidence of a direct causal pathway. An unexpected pattern was observed for household income in the fully adjusted model. Although lower income was associated with a higher prevalence of depression in the bivariate analysis, several income categories showed attenuated or reversed associations after full adjustment for emotional and environmental factors. This reversal may reflect statistical suppression or shared variance with related socioeconomic and health-status indicators included in the model.

## 4. Discussion

### 4.1. Principal Findings

This study shows that when lifetime diagnosed depression among adults with diagnosed diabetes is examined through a theory-driven, population-level framework, emotional and environmental conditions emerge as the strongest correlates, outweighing individual health behaviors in fully adjusted models. Frequent mental distress showed a very strong association with lifetime diagnosed depression, while social and economic stress and poor self-rated health remained independently associated after adjusting for sociodemographic and behavioral factors, highlighting the strong association of sustained emotional burden and contextual strain. The attenuation of behavioral associations in the full model suggests that commonly targeted diabetes self-management behaviors may be more closely linked to underlying emotional and environmental influences than to depression independently. By applying the MTM to nationally representative surveillance data, this study demonstrates how MTM domains can help distinguish emotional and contextual correlates from behavioral factors, strengthening the translational relevance of MTM for public health research and practice [14,15,25]. Because frequent mental distress captures recent emotional symptoms that may overlap with depressive experiences, the strong association observed likely reflects shared emotional burden rather than a purely independent predictor–outcome relationship.

### 4.2. Interpretation Through the MTM

Within the MTM framework, the prominence of emotional and environmental factors in the fully adjusted model is best understood through the construct of emotional transformation, which emphasizes the capacity to regulate and redirect emotional experiences in ways that sustain health-related functioning under chronic stress. In this study, frequent mental distress represents a population-level indicator of compromised emotional transformation, reflecting persistent negative affect that undermines coping capacity, motivation, and perceived control. Among adults managing diagnosed diabetes, ongoing emotional strain related to symptom burden, treatment demands, and fear of complications may overwhelm efforts to maintain emotional equilibrium, thereby increasing vulnerability to depression independent of individual health behaviors [4,14,15]. The strength of the observed association for mental distress underscores MTM’s premise that emotional processes are not peripheral correlates but important sustaining mechanisms shaping mental health outcomes in chronic disease contexts. Environmental context further clarifies why social and economic stress and poor self-rated health remained robust correlates of depression. MTM conceptualizes the social environment as a sustaining force that can either support or undermine long-term health outcomes. Elevated socioeconomic stress may restrict access to supportive resources, intensify daily demands, and limit opportunities for recovery, thereby perpetuating depressive states even when individuals engage in recommended health behaviors [18,19]. Similarly, fair or poor perceived health likely reflects an accumulation of physical limitations, comorbid conditions, and perceived loss of functioning, reinforcing negative emotional appraisals and diminishing confidence in one’s ability to manage diabetes over time [11]. These findings align with MTM’s emphasis on environmental conditions as necessary support for sustaining positive health outcomes. The attenuation of behavioral factors in the full model should be interpreted within this theoretical context. Behaviors such as physical activity, smoking, and body weight may function as downstream expressions of emotional burden and adverse environmental conditions rather than as independent correlates of depression. When emotional distress and contextual strain are unaccounted for, behavioral associations appear salient; however, once these upstream MTM domains are included, behavioral effects diminish, suggesting shared variance or indirect pathways through emotional and environmental processes. This pattern does not indicate that health behaviors are unimportant but rather that their association with depression risk is strongly context dependent and embedded within broader emotional and social conditions, consistent with MTM’s distinction between initiation and sustenance processes [11,14,15,16]. This interpretation is further supported by prior research demonstrating that depression often precedes declines in diabetes self-care behaviors rather than arising solely as a consequence of poor behavioral adherence [7,26]. From an MTM perspective, sustained behavior change is unlikely in the absence of emotional transformation and supportive environments, explaining why behavior-focused models alone may inadequately capture depression risk among adults with diagnosed diabetes [15,27].

### 4.3. Comparison with Prior Studies

The present findings align with the broader diabetes–depression literature, showing that depression is not only common among adults with diabetes but is also closely linked to functional burden, poorer perceived health, and barriers to effective self-management. Meta-analytic evidence has consistently demonstrated that diabetes is associated with substantially higher odds of comorbid depression across diverse populations and study designs [28]. Previous research also indicates that depression is associated with worse diabetes outcomes through mechanisms such as reduced adherence and diminished engagement in self-care, rather than through isolated health behaviors alone [16]. Similarly, reviews emphasize that comorbid depression in diabetes is linked to increased morbidity, disability, and health care utilization, highlighting the clinical and public health importance of identifying modifiable and context-sensitive correlates. The present study extends these findings by demonstrating that when emotional and environmental conditions are examined alongside behavioral factors, emotional distress and contextual strain remain dominant correlates. This pattern supports the view that depression is closely linked to the lived experience of chronic disease, including complications, perceived health decline, and exposure to psychosocial stressors [10,16].

This study also contributes to the expanding literature applying the MTM by illustrating how its domains can be used to interpret mental health outcomes within a chronic disease framework. Prior MTM research has largely focused on predicting the initiation and maintenance of specific health behaviors, consistently emphasizing the importance of emotional and environmental mechanisms in sustaining behavior change [15,29,30]. For example, MTM-based investigations in diverse behavioral areas highlight that sustained change depends strongly on emotional and social/environment mechanisms, supporting MTM’s emphasis on emotional transformation and environmental support as key components of maintenance rather than optional add-ons [15,30]. In contrast to much of the prior MTM literature, which typically treats mental health as a background characteristic or secondary covariate, the present analysis places depression as the primary outcome and shows that emotional and environmental domains account for the strongest observed associations with depression once behavioral factors are considered. This represents a meaningful theoretical contribution for public health journals because it shows that MTM can guide interpretation beyond behavior-only models by clarifying why behavioral associations may weaken in fully adjusted analyses: behaviors may reflect downstream consequences of emotional burden and constrained environments rather than independent correlates. Taken together, the comparison across studies positions this study as an integrative contribution that bridges diabetes epidemiology and theory-driven public health research, providing a clearer roadmap for intervention priorities that extend beyond lifestyle counseling to include distress-focused and context-responsive strategies.

The inverse association observed for lower household income in the fully adjusted model contrasts with both the bivariate results and prior research, which generally link socioeconomic disadvantage with higher depression risk. One plausible explanation is statistical suppression resulting from the inclusion of closely related variables such as social or economic stress and self-rated health, which may capture overlapping aspects of socioeconomic vulnerability. When these correlated indicators are included simultaneously in the model, the remaining independent effect of income may appear attenuated or reversed.

### 4.4. Public Health and Clinical Implications

The findings of this study have direct implications for public health practice and diabetes care by underscoring the need to integrate mental health more fully into routine diabetes management rather than treating it as a secondary or optional concern. The strong and independent associations observed for emotional distress and adverse social conditions indicate that depression among adults with diagnosed diabetes cannot be adequately addressed through behavior-focused counseling alone. Public health strategies that prioritize glycemic control and lifestyle modification without concurrent attention to emotional well-being and contextual stressors may miss important correlates of depression risk. Integrating mental health services into diabetes care, including collaborative care models and coordinated screening pathways, has been shown to improve both psychological outcomes and diabetes-related functioning, supporting a more holistic and sustainable approach to chronic disease management [26]. Routine screening for psychological distress and social stressors represents a practical and actionable implication of these findings. Measures such as frequent mental distress and perceived stress capture ongoing emotional strain that may not meet diagnostic thresholds for major depression but may identify individuals at elevated risk. Population-based guidelines increasingly emphasize the importance of routine depression screening in adults with chronic disease, particularly when symptoms may interfere with self-care and treatment engagement [31,32]. Incorporating brief distress and social needs screening into diabetes care settings can facilitate early identification of individuals at heightened risk and support timely referral to mental health or social services. From a public health perspective, this approach aligns with calls to address social determinants of health as integral components of chronic disease prevention and management rather than as external or downstream concerns [18,19].

The MTM provides a useful framework for translating these implications into intervention design by emphasizing emotional transformation and supportive environments as prerequisites for sustained health outcomes. MTM-informed interventions can move beyond education and skill-building to target emotional regulation, coping capacity, and environmental supports that enable individuals to manage diabetes in the context of ongoing stress. For example, interventions that incorporate stress management, problem-solving skills, and social support enhancement may better address the emotional and contextual correlates of depression identified in this study. Applying MTM to public health and clinical interventions may therefore improve effectiveness by aligning strategies with the domains most strongly associated with depression risk, offering a theory-guided pathway for integrating mental health promotion into diabetes prevention and care efforts [14,25].

### 4.5. Strengths and Limitations

The key strengths and limitations of this study are summarized in Table 4.

### 4.6. Future Research Directions

Future research should extend these findings using longitudinal designs to clarify how emotional distress, environmental pressures, and health behaviors interact over time to shape depression risk among adults with diagnosed diabetes. Such studies would provide clearer insight into the temporal dynamics of MTM-related processes, particularly emotional transformation and environmental context, within chronic disease populations. Longitudinal evidence could also help determine whether emotional distress precedes behavioral challenges or develops alongside them. Future work may also benefit from testing MTM-informed interventions that address emotional regulation, social stressors, and environmental supports in order to evaluate whether these domains can improve mental health outcomes in diabetes care [33].

### 4.7. Implications for Theory and Practice

The findings contribute to theory by reinforcing the utility of the MTM as a framework for understanding mental health outcomes within chronic disease contexts, particularly by highlighting the stronger observed role of emotional and environmental domains. For practice, the results support the integration of routine screening for distress and social stressors into diabetes care and public health programming, rather than focusing exclusively on lifestyle behaviors. MTM-informed interventions that explicitly address emotional burden and environmental constraints alongside self-management behaviors may offer more effective and sustainable approaches to reducing depression risk and improving quality of life among adults with diagnosed diabetes.

## 5. Conclusions

Applying the MTM of Health Behavior Change showed that emotional distress and adverse social contexts were more strongly associated with depression among adults with diagnosed diabetes than individual health behaviors in fully adjusted models. These findings underscore the importance of moving beyond behavior-only approaches in diabetes care and adopting integrated, theory-guided strategies that address emotional burden and contextual stressors as important components of mental health promotion. From a public health perspective, incorporating routine distress screening and context-sensitive support into diabetes prevention and management may enhance both mental health outcomes and sustained self-management.

## Figures and Tables

**Table 1:** Weighted descriptive characteristics of U.S. adults with diagnosed diabetes (*n* = 19,967), BRFSS 2023.

Characteristic	Category	Unweighted N	Weighted %
Sex	Female	10,406	50.5
	Male	9663	49.5
Age group (years)	18–24	37	0.4
	25–29	58	0.8
	30–34	148	1.6
	35–39	296	2.7
	40–44	476	4.3
	45–49	836	5.8
	50–54	1277	9.5
	55–59	1850	11.0
	60–64	2679	15.6
	65–69	3260	13.5
	70–74	3376	14.2
	75–79	2846	10.4
	≥80	2589	10.1
Race/Ethnicity	White, non-Hispanic	12,874	63.8
	Black, non-Hispanic	2846	12.1
	Hispanic	2213	14.7
	Asian, non-Hispanic	694	4.3
	American Indian/Alaska Native	821	3.2
	Other/Multiracial	519	1.9
Educational attainment	<High school	2941	15.4
	High school graduate	6214	30.7
	Some college/technical school	6947	34.8
	College graduate	3865	19.1
Household income	<$15,000	3428	18.2
	$15,000–$24,999	3901	20.4
	$25,000–$34,999	3012	14.8
	$35,000–$49,999	3256	15.9
	$50,000–$74,999	3041	15.3
	≥$75,000	3329	15.4
Self-rated general health	Excellent	1624	7.8
	Very good	3908	18.9
	Good	6841	34.1
	Fair	4796	25.1
	Poor	2798	14.1

Table Note: Values are presented as unweighted sample counts (N) and survey-weighted percentages (%). Percentages represent weighted estimates using BRFSS 2023 final sampling weights and account for the complex survey design. Percentages may not amount to 100 due to rounding or missing data.

**Table 2:** Survey-weighted bivariate associations between depression and study variables among U.S. adults with diagnosed diabetes, BRFSS 2023.

Characteristic	Category	Depression (%)	No Depression (%)	Rao–Scott χ^2^	*p*-Value
Sex	Female	27.6	72.4	6.8	0.009
	Male	21.0	79.0		
Age group	Younger adults	29.4	70.6	32.1	<0.001
	Older adults	22.0	78.0		
Race/Ethnicity	White, non-Hispanic	25.1	74.9	9.7	0.021
	Black, non-Hispanic	20.3	79.7		
	Hispanic	21.5	78.5		
Education	<High school	33.2	66.8	41.6	<0.001
	≥Some college	21.4	78.6		
Household income	Lower income	34.8	65.2	45.9	<0.001
	Higher income	18.7	81.3		
Health insurance	Uninsured	32.6	67.4	7.4	0.007
	Insured	23.9	76.1		
Self-rated general health	Fair/Poor	41.3	58.7	128.5	<0.001
	Good–Excellent	14.9	85.1		
Physical activity	No	31.2	68.8	45.7	<0.001
	Yes	18.4	81.6		
Smoking status	Current	30.1	69.9	6.1	0.014
	Never	22.5	77.5		
Alcohol use	Yes	27.8	72.2	4.9	0.027
	No	22.6	77.4		
Frequent mental distress	Yes	67.9	32.1	412.6	<0.001
	No	13.2	86.8		
Social/Economic stress	High	39.6	60.4	96.8	<0.001
	Low	19.1	80.9		

Table note: Values are presented as survey-weighted percentages. Bivariate associations were evaluated using Rao–Scott χ^2^ tests to account for the complex BRFSS sampling design. Percentages may not amount to 100 due to rounding. Statistical significance was assessed at α = 0.05. Categories labeled as “lower” and “higher” income represent collapsed income groups (<$35,000 vs. ≥$75,000).

**Table 3:** MTM-guided survey-weighted logistic regression models examining depression among U.S. adults with diagnosed diabetes, BRFSS 2023.

Predictor	Model 1 AOR (95% CI)	*p*	Model 2 AOR (95% CI)	*p*	Model 3 AOR (95% CI)	*p*
Age group (vs. 60–64)						
Age 18–24	4.97 (2.25–10.99)	<0.001	5.15 (2.24–11.84)	<0.001	7.56 (0.04–1510.43)	0.454
Age 25–29	2.15 (0.83–5.59)	0.113	2.09 (0.79–5.54)	0.138	1.57 (0.15–16.39)	0.704
Age 30–34	2.18 (1.21–3.93)	0.010	2.03 (1.10–3.75)	0.024	2.73 (0.81–9.17)	0.104
Age 35–39	1.16 (0.59–2.28)	0.663	1.15 (0.58–2.27)	0.683	0.27 (0.09–0.83)	0.023
Age 40–44	1.49 (1.00–2.20)	0.049	1.43 (0.95–2.16)	0.084	2.00 (0.75–5.34)	0.165
Age 45–49	1.27 (0.83–1.95)	0.270	1.21 (0.77–1.90)	0.403	1.23 (0.41–3.68)	0.707
Age 50–54	1.15 (0.79–1.69)	0.465	1.11 (0.74–1.66)	0.603	1.27 (0.39–4.11)	0.699
Age 55–59	1.12 (0.77–1.64)	0.544	1.09 (0.73–1.63)	0.674	2.06 (0.79–5.37)	0.139
Age 65–69	0.91 (0.63–1.32)	0.622	0.93 (0.63–1.36)	0.709	0.80 (0.33–1.94)	0.632
Age 70–74	0.80 (0.54–1.19)	0.266	0.80 (0.53–1.20)	0.279	0.63 (0.27–1.47)	0.304
Age 75–79	0.72 (0.46–1.12)	0.144	0.73 (0.46–1.15)	0.177	0.29 (0.10–0.84)	0.027
Age 80–84	0.66 (0.40–1.09)	0.105	0.68 (0.40–1.14)	0.141	0.56 (0.13–2.37)	0.443
Age ≥ 85	0.67 (0.35–1.28)	0.227	0.68 (0.35–1.31)	0.249	0.72 (0.09–5.86)	0.747
Sex						
Male (vs. Female)	0.39 (0.34–0.45)	<0.001	0.43 (0.36–0.50)	<0.001	0.70 (0.44–1.11)	0.142
Race/Ethnicity (vs. White)						
Black	0.49 (0.36–0.67)	<0.001	0.49 (0.32–0.74)	<0.001	0.67 (0.37–1.21)	0.177
Asian	0.35 (0.18–0.67)	0.001	0.44 (0.20–0.96)	0.040	0.73 (0.33–1.62)	0.461
American Indian/Alaska Native	0.74 (0.40–1.37)	0.338	0.80 (0.40–1.61)	0.540	0.57 (0.25–1.32)	0.190
Native Hawaiian/Other Pacific Islander	1.20 (0.62–2.33)	0.590	1.42 (0.66–3.07)	0.370	3.47 (1.74–6.93)	<0.001
Other race	1.28 (0.81–2.02)	0.292	1.30 (0.77–2.20)	0.327	2.01 (1.15–3.52)	0.014
Behavioral Factors						
Physical activity (Yes vs. No)	—	—	0.78 (0.60–0.99)	0.044	1.14 (0.78–1.66)	0.495
BMI (per 1 unit increase)	—	—	1.05 (1.04–1.06)	<0.001	1.01 (0.99–1.03)	0.911
Former smoker (vs. Never)	—	—	1.18 (0.96–1.46)	0.119	1.09 (0.86–1.39)	0.476
Current smoker (vs. Never)	—	—	1.31 (1.08–1.60)	0.006	1.36 (0.91–2.03)	0.144
Alcohol use (Yes vs. No)	—	—	0.74 (0.63–0.87)	<0.001	0.74 (0.58–0.95)	0.016
Emotional Factor						
Frequent mental distress (≥14 days)	—	—	—	—	10.36 (7.57–14.19)	0.002
Environmental Factors						
High social/economic stress	—	—	—	—	2.56 (1.88–3.49)	<0.001
Fair/Poor general health	—	—	—	—	3.61 (1.49–8.74)	0.005
Health insurance (Yes vs. No)	—	—	—	—	0.64 (0.41–1.00)	0.052
Education (vs. High school graduate)						
≤High school	—	—	—	—	1.35 (0.90–2.02)	0.141
Some college+	—	—	—	—	1.28 (0.90–1.82)	0.164
Income (vs. $35–50 k)						
<$10 k	—	—	—	—	1.12 (0.39–3.20)	0.833
$10–15 k	—	—	—	—	0.70 (0.35–1.41)	0.316
$15–20 k	—	—	—	—	0.80 (0.45–1.41)	0.454
$20–25 k	—	—	—	—	0.81 (0.48–1.36)	0.409
$25–35 k	—	—	—	—	0.95 (0.55–1.63)	0.848
$50–75 k	—	—	—	—	0.77 (0.47–1.27)	0.327
$75–100 k	—	—	—	—	0.52 (0.26–1.04)	0.060
$100–150 k	—	—	—	—	0.31 (0.15–0.65)	<0.001
$150–200 k	—	—	—	—	0.54 (0.26–1.13)	0.090
>$200 k	—	—	—	—	0.68 (0.32–1.46)	0.347

Table Note: Values are adjusted odds ratios (AORs) with 95% confidence intervals (CIs) derived from survey-weighted logistic regression models accounting for BRFSS sampling weights, stratification, and primary sampling units. Model 1 adjusted for sociodemographic characteristics (age group, sex, and race/ethnicity). Model 2 additionally adjusted for behavioral factors including physical activity, body mass index, smoking status, and alcohol use. Model 3 further adjusted for emotional and environmental factors including frequent mental distress, social/economic stress, self-rated general health, education, household income, and health insurance.

**Table 4:** Summary of the strengths and limitations of the present study based on BRFSS 2023 data

Domain	Description
Strengths	
National representativeness	The study used a large, population-based sample derived from nationally available BRFSS data, allowing findings to be generalized to U.S. adults with diagnosed diabetes rather than restricted clinical or regional cohorts.
Theory-driven analytic framework	The study explicitly applied the MTM of Health Behavior Change as an organizing framework, enabling systematic examination of behavioral, emotional, and environmental domains rather than relying on atheoretical or behavior-only models.
Integrated modeling approach	Nested survey-weighted regression models were used to empirically assess the relative contribution of MTM domains, allowing for the identification of dominant emotional and environmental pathways while accounting for behavioral and sociodemographic factors.
Public health relevance	Findings translate complex theoretical constructs into measurable population-level indicators, strengthening the applicability of MTM for public health surveillance, intervention design, and policy-relevant decision making.
Limitations	
Cross-sectional design	The cross-sectional nature of the data prevents assessment of temporal relationships, and causal inferences between depression and associated factors cannot be established.
Self-reported measures	All variables were based on self-reports, which may introduce recall bias or misclassification, particularly for behavioral measures such as physical activity, smoking, and alcohol use.
Outcome measurement	Depression was assessed as lifetime diagnosed depression rather than current symptom severity, which may not fully capture fluctuations in mental health status over time. Frequent mental distress reflects recent psychological strain that may overlap with symptoms of depression. Therefore, the strong association observed in this study may partly reflect shared emotional burden rather than a completely independent explanatory factor.
Temporal ambiguity of depression diagnosis	Depression was measured as lifetime diagnosed depression rather than current depressive symptoms. Because BRFSS does not capture when the diagnosis occurred relative to the onset of diabetes, the associations observed should be interpreted as correlates of comorbid depression history rather than indicators of temporal or causal relationships.
Residual confounding	Although several behavioral, emotional, and environmental factors were included, some relevant clinical variables were not available in the BRFSS dataset. These include measures of diabetes duration and severity (e.g., HbA1c or complications), treatment modality (insulin versus oral medication), coexisting chronic conditions, and prior depression treatment. In addition, while socioeconomic stressors were considered, the survey did not include a direct measure of social support, an important component of the social environment within MTM. Therefore, residual confounding cannot be completely excluded.

## Data Availability

The data presented in this study are available from the U.S. Centers for Disease Control and Prevention (CDC) Behavioral Risk Factor Surveillance System (BRFSS) at https://www.cdc.gov/brfss (accessed on 26 December 2025). These data were derived from resources available in the public domain, specifically the 2023 BRFSS public-use dataset provided by the CDC.

## Abbreviations

The following abbreviation is used in this manuscript:

AOR	Adjusted Odds Ratio
BRFSS	Behavioral Risk Factor Surveillance System
CDC	Centers for Disease Control and Prevention
CI	Confidence Interval
IRB	Institutional Review Board
MTM	Multi-Theory Model
PSU	Primary Sampling Unit
STROBE	Strengthening the Reporting of Observational Studies in Epidemiology
